# Survival analysis of young adults from a Brazilian cohort of non-small cell lung cancer patients

**DOI:** 10.3332/ecancer.2021.1279

**Published:** 2021-08-24

**Authors:** Jéssica Silva Nicolau, Rossana Veronica Mendoza Lopez, Carolina Terra de Moraes Luizaga, Karina Braga Ribeiro, Rosimeire Aparecida Roela, Simone Maistro, Maria Lucia Hirata Katayama, Renato José Mendonça Natalino, Gilberto de Castro, Jose Eluf Neto, Maria Aparecida Azevedo Koike Folgueira

**Affiliations:** 1 Instituto do Câncer do Estado de São Paulo (ICESP), Hospital das Clinicas HCFMUSP, Faculdade de Medicina, Universidade de Sao Paulo, Sao Paulo, SP, 01246-000, Brazil; 2 Fundação Oncocentro de Sao Paulo (FOSP), Sao Paulo, SP, 05409-012, Brazil; 3 Faculdade de Ciências Médicas da Santa Casa de Sao Paulo, Sao Paulo, SP, 01238-010, Brazil; 4 Departamento de Medicina Preventiva, Faculdade de Medicina, Universidade de Sao Paulo, Sao Paulo, SP, 01246 903, Brazil; † JSN and RVML contributed equally to this work.

**Keywords:** lung carcinoma, non-small cell lung cancer, young adult, survival, clinical features

## Abstract

**Background:**

The influence of age at diagnosis in non-small cell lung cancer (NSCLC) prognosis is unclear.

**Objectives:**

To compare in a Brazilian cohort of NSCLC patients of different age groups: 1) The overall survival; 2) Clinical features and treatment options.

**Methods:**

This is a retrospective cohort study using a hospital-based registry, for NSCLC patients registered in years 2000–2009. Patients were grouped into three age groups: Young adults (YA: < 40 years), middle-aged (MA: 40–64 years) and elderly (E: ≥ 65 years). Kaplan–Meier was used to estimate overall survival and Cox regression for hazard ratios (HRs) and 95% confidence intervals.

**Results:**

17,422 NSCLC patients were included: 370 YA (2.1%), 8,697 MA (49.9%) and 8,355 E (48.0%). Compared with older age groups, the YA group had a higher proportion of females, patients diagnosed with adenocarcinoma and metastatic disease (63.2%). Overall survival was longer in YA in the entire cohort and in all clinical stages (CSs) (p < 0.001). For YA, higher education level was a good prognosis factor (compared with illiterate and incomplete elementary); advanced or metastatic disease (compared with early-stage disease) and treatment based in radiotherapy or chemotherapy (CT) (without surgery), compared with treatment combinations with surgery, were poor prognostic factors. Young men (but not women) had lower HR of death compared with older groups; YA had lower HR of death in all CSs compared with patients from older groups. A higher percentage of YA were treated with surgery or CT in early-stage disease compared with older groups. Besides that, YA and MA patients treated with surgery or CT had a better prognosis than elderlies. Conclusions: In this Brazilian cohort of NSCLC patients, most young individuals were diagnosed with metastatic disease. YA presented longer survival than older age groups in all CSs, but mainly in CS I/II and III, where some patients may achieve long remissions or cure.

## Introduction

Lung cancer is one of the deadliest cancers worldwide. In Brazil, lung cancer is one of the most common cancers and according to INCA (Instituto Nacional do Câncer – Brazilian National Cancer Institute) [1], 17,760 new cases in men and 12,440 new cases in women were estimated for 2020. Survival rates are highly dependent on clinical stage at diagnosis. In the USA, five-year relative survival rates for the years 2008 to 2014, varied from 56% for patients with localized disease to 30% and 5% for patients with regional and metastatic disease, respectively. Unfortunately, as much as 57% of the patients are diagnosed with metastatic disease and only 16% and 22% are diagnosed with localized and regional disease, respectively [2].

Lung cancer is usually diagnosed in people older than 60 years. There is data showing that age-specific incidence rates of lung cancer rise steeply from around age 45 to 49, to reach the highest rates in the 80–84 age group for females and the 85–89 age group for males, in the United Kingdom [3], as well as SSER showing that most patients are diagnosed with lung cancer at ages 60–69 years (37%), followed by 50–59 years (25.2%) and 70–79 years (22.9%) in the USA. Nonetheless, 8.99%–11.6% of the cases occur in individuals with ages below 51 years [4, 5].

Some studies have compared clinical features of lung cancer among young and elderly individuals. In the younger age group, the frequency of women, African Americans and adenocarcinoma was higher [4–6]. There is also data showing that younger patients are more frequently diagnosed with metastatic disease, as compared to older patients [5].

In Brazil, an ecological study using population-based cancer registries, hospital-based cancer registries and the national mortality database, for the period 2000–2014, revealed that almost 60% of the lung cancer patients presented in the age interval of 50–69 years, most were males (63.5%) and diagnosed with adenocarcinoma histology (41.9%) [7]. Among younger patients, aged 18–49 years, a larger proportion of women was detected. Unfortunately, more than 50% of the patients were diagnosed with metastatic disease and more than 30% in clinical stage (CS) III. This study showed that both incidence and mortality rates for women with lung cancer increased in more recent years [7].

There is conflicting data regarding the influence of age at diagnosis on lung cancer prognosis. It was reported that young patients with inoperable tumours have a shorter survival as compared to a reference group of older patients [8]. Some studies, however, found no age-related differences on survival [9–11], while others reported a longer survival for young adults (YA) compared with older age groups [4–6, 12, 13]. However, the cutoff age varied from 40 [5] to 50 years [14] and even though some studies have only evaluated non-small cell lung cancer (NSCLC) patients [5, 14], others have included both SCLC and NSCLC [4, 6, 11]. Some of these studies attributed the survival advantage in young patients to more aggressive treatments and a higher incidence of somatic mutations in therapy target genes, such as Epidermal growth factor receptor (*EGFR)* [4, 5].

In Brazil, lung cancer represents a great challenge. Patients are frequently diagnosed with advanced stage disease, probably due to a delay in diagnosis. In addition, there is a low frequency of patients receiving curative-intent therapy and molecular testing is not yet available to all patients [15]. Besides that, there is no data specifically evaluating young patients.

Hence, the main goal of the present study was to evaluate overall survival for YA, diagnosed with NSCLC, compared with older age groups, in a cohort of patients from Sao Paulo State, Brazil. The secondary goal was to compare clinical features and treatment options in different age groups.

## Material and methods

A retrospective cohort study was performed using the hospital-based registry from Fundação Oncocentro de Sao Paulo (FOSP) [16], which gathers data from public hospitals, as well as from some private and philanthropic hospitals from Sao Paulo State, Brazil.

The inclusion criteria were (i) diagnosis of malignant neoplasm of the lung (International Classification of Diseases for Oncology, C34) in the period between 2000 and 2009; (ii) lung as the primary site; (iii) age ≥ 18 years at diagnosis. The exclusion criteria were (i) diagnosis of small cell carcinoma, neuroendocrine tumour, atypical neoplasm, carcinoma *in situ*, rare tumours (less than ten cases) in the dataset; (ii) null follow-up; (iii) inconsistency in dates of diagnosis and death (negative follow-up). Both ii and iii were similarly distributed in the three age groups, with an incidence of less than 1.0%.

**Figure 1.**
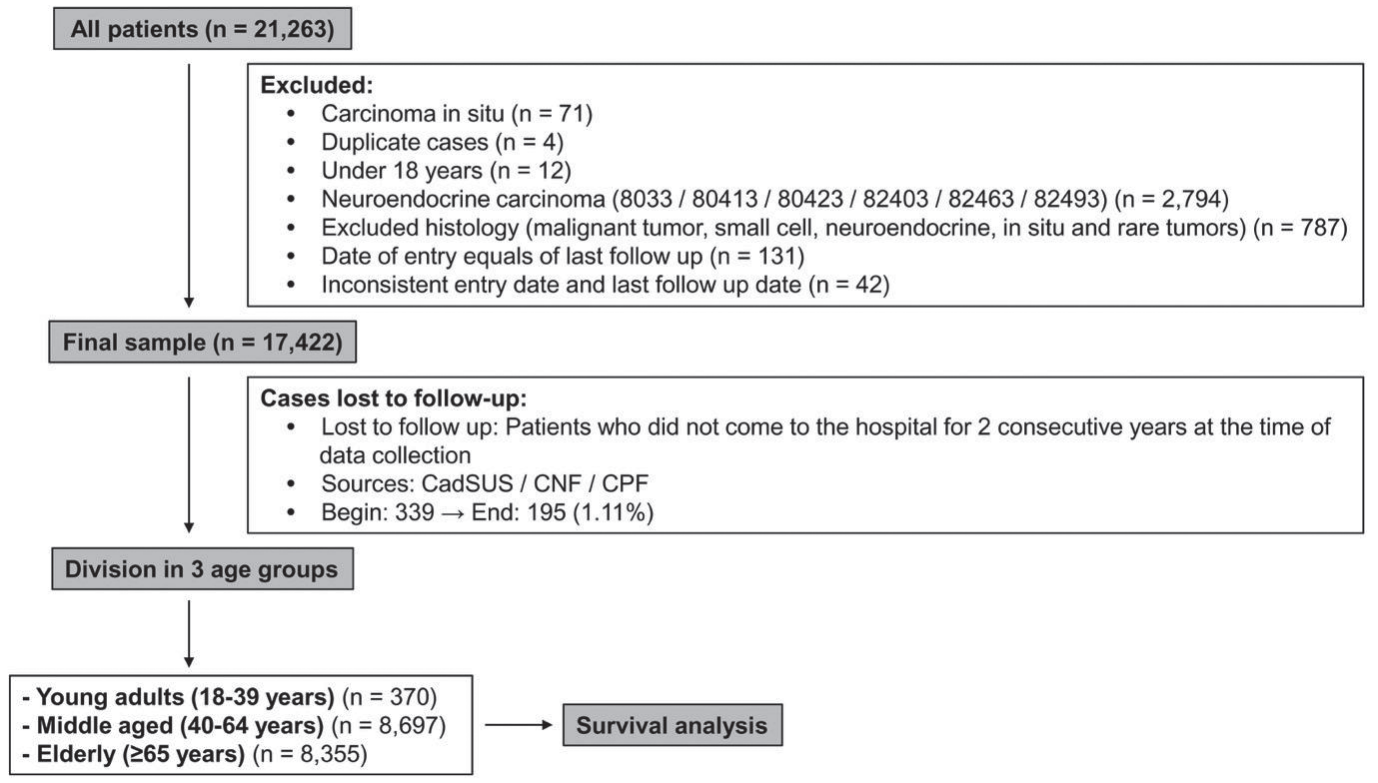
Summary of case selection, case exclusion and division on three age groups.

Data were retrieved from 21,263 patients and the studied sample consisted of 17,422 patients, after exclusions due to inclusion and exclusion criteria. Data included information on sex, histological type, CS, date of diagnosis and last date of follow-up. Data were collected on a yearly basis, thus lost to follow-up was defined for those living patients that did not come to the hospital for 2 consecutive years, at the time of the latest data collection. For these patients, another attempt to establish their vital status consisted of the search of other public databases: Unified Health User Registration System (CadSUS), National Register of the Deceased (CNF) and Individual Entity Registry (CPF). In CadSUS and CNF, the information obtained was the patient date of death, whereas in the CPF consultation, only the year of death was obtained, and 1 July was standardised as the last follow-up date, for all cases found in a specific year. In the end of this search, 195 cases remained as lost to follow-up (1.11%) (Figure 1).

For this study, YA were those patients diagnosed with cancer between 18 and 39 years old [17]. Elderly patients (E) were those aged 65 or over (‘World Population Ageing 2019 Highlights’, United Nations, Department of Economic and Social Affairs, Population Division (2019) – World Population Ageing 2019: Highlights) [18] and middle-aged (MA) were patients aged 40–64 years.

Clinical staging, based in the American Joint Committee on Cancer (AJCC) Cancer Staging, was retrieved from the database. In the period of the study (2000–2009), both AJCC 5th (1997) and 6th (2002) editions were effective; however, lung cancer staging criteria were kept unchanged in these editions.

Descriptive statistics were calculated for all variables. Mean, median, standard deviation and minimum and maximum values are shown for quantitative variables. Qualitative variables are shown as frequencies and proportions. Pearson’s chi-squared test was used to determine whether there was a statistically significant difference between the expected frequencies and the observed frequencies in demographic variables, disease characteristics and treatment types (surgery: yes versus no; radiotherapy (RT): yes versus no; chemotherapy (CT): yes versus no).

Overall survival was calculated in months from date of diagnosis until date of death, or last information. Patients alive or lost to follow-up were censored (18 August 2019, was the end of follow-up). Survival curves were performed using the Kaplan–Meier method and compared with the log-rank test.

Hazard ratios (HRs) and 95% confidence intervals (95% CI) were calculated using Cox regression model for demographic variables, including sex (women and men), education (illiterate, incomplete elementary, complete elementary, high school and university education); disease characteristics, including tumour histology (squamous cell, adenocarcinoma, large cells, and other unspecified), CS (I/II, III and IV); treatment (surgery: alone or combined with RT, CT, other combinations; CT alone or combined with RT; RT; other combinations; no treatment). Level of significance was two-sided 5% for all hypothesis tests. Analyses were performed using SPSS v.18 for Windows or Stata/MP 14.0 for Windows.

## Results

### Characteristics of the study population

**Supplementary Figure 1.**
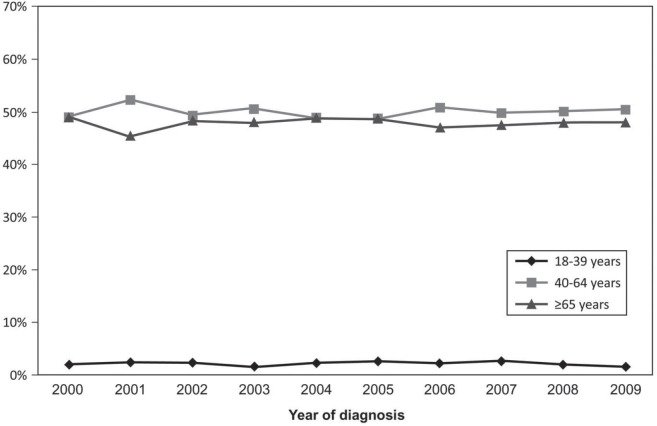
Percentage of cases according to the age group and to the year of diagnosis.

The population of the study consisted of 17,422 patients diagnosed with NSCLC in the period from 2000 to 2009: 370 YA (YA: 2.2%), 8,697 MA (MA: 49.9%) and 8,355 elderlies (E: 48.0%) (Table 1). The median age at diagnosis was 36 (YA), 56 (MA) and 72 years (E). The median and mean follow-up times were 7.95 months (minimum: 0.03–maximum: 220.22) and 17.47 months (SD: 27.55 months). Initially, we evaluated the percentage of patients diagnosed with NSCLC in each year of the 2000–2009 interval, according to the age groups, and YA consisted of 1.5%–2.7% along the years (Supplementary Figure 1).

Although among YA, almost half of the patients were females (48.1% versus 51.9% males), in MA and elderlies, most patients were males (MA: 63.9%; E: 71.5%, *p* < 0.001).

Regarding the education level, information was available for approximately 75% of the patients, in each age group. In the whole group, only 7.2% reported University education. Besides that, there was a statistical difference among age groups and while more YA completed high school (29.5%), illiteracy was predominant in the elderly group (15.0%) (*p* < 0.001) (Table 1). Considering the histological type, adenocarcinoma was relatively more frequent, while squamous cell carcinoma was less frequent in the group of YA (*p* < 0.001). CS at diagnosis revealed a higher frequency of lung cancer diagnosed as metastatic disease (stage IV) in the group of YA (YA: 63.2% versus MA: 51.9% versus E: 43.2%, *p* < 0.001) (Table 1).

The main forms of treatment offered to patients, including surgery (yes versus no); RT (yes versus no) and CT (yes versus no) were analysed in the different age groups. The results revealed that a lower percentage of elderly patients received the main forms of treatment (compared with younger age groups) (Table 1)**.** We further dissected this data, considering treatment within the CSs and observed that a higher percentage of young patients were treated with surgery in CSs I/II and III and with CT in CS I/II (than patients in older age groups). On the other hand, a lower percentage of elderly patients received surgery and CT in all CSs, but a higher percentage received RT in CSs I/II (than younger age groups) (Figure 3).

### Survival analysis

Among 17,422 patients considered for the analysis, 16,639 had died at the end of the observation period (335 YA, 8,213 MA and 8,091 elderly). The probability of survival in 1, 2, 5 and 10 years was 37.5%, 18.9%, 7.4% and 3.8% for the whole group of patients (Table 2).

**Figure 2.**
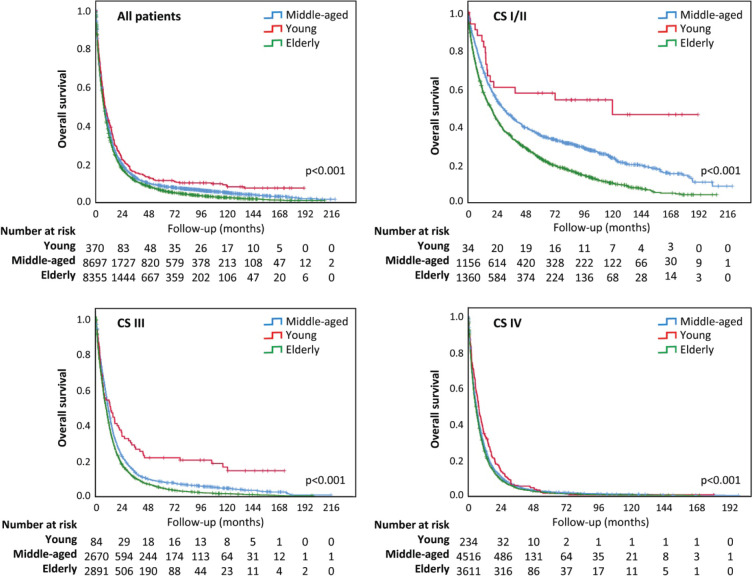
Overall survival according to age group. (a): Survival analysis for all patients; (b): Survival analysis for early CS (I/II); (c): Survival analysis for CS III; (d): Survival analysis for CS IV.

**Figure 3.**
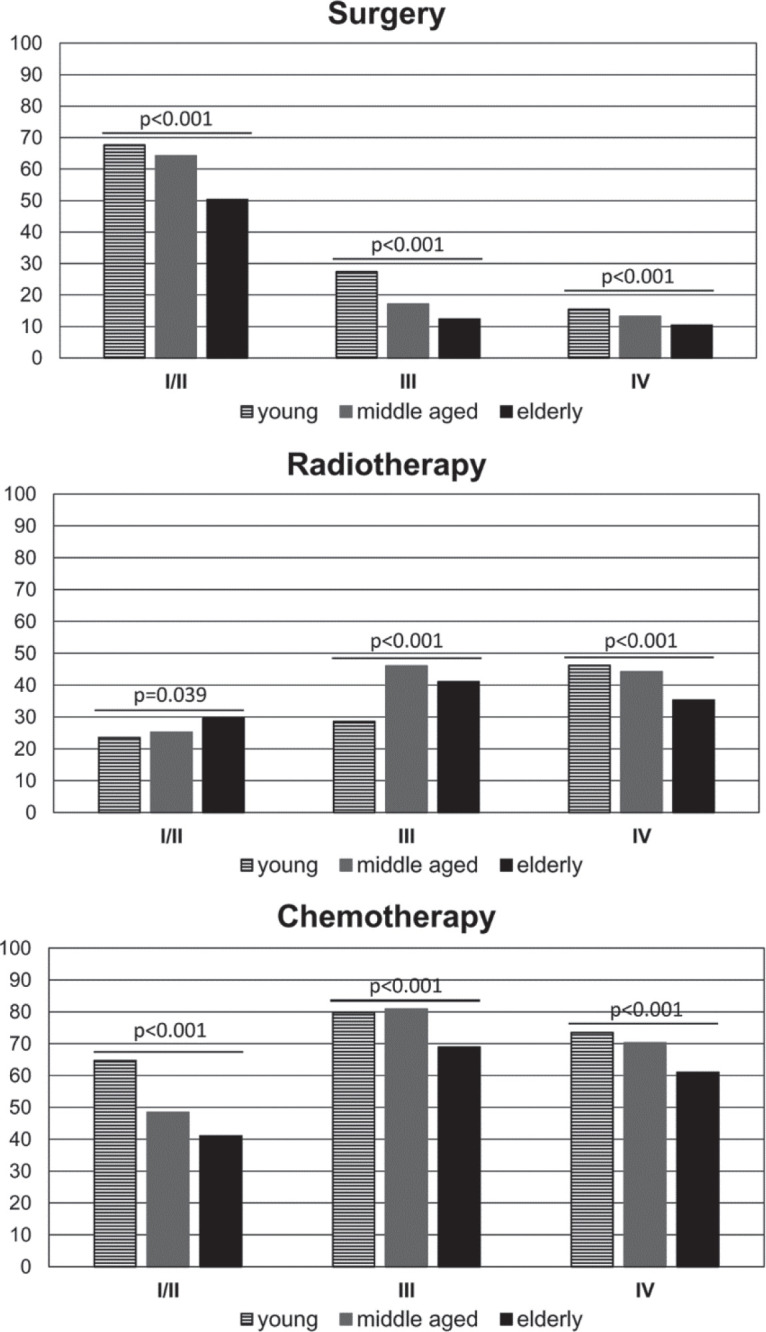
Percentage of patients treated with surgery, RT or CT according to the CS (CS I/II; III and IV) and the age group.

Overall survival was different among age groups, longer for YA (*p* < 0.001) (Figure 2). The median survival time was 7.98 months for all lung cancer patients and 8.41, 8.51 and 7.49 months for YA, MA and elderly people, respectively. Although the overall survival curves have runed together in the first months of follow-up, they distanced thereafter (Figure 2). In addition, YA had a longer overall survival in all the CSs (I/II, III and IV) (Figure 2).

### HR of death – univariate and multivariable analysis

We evaluated the HR of death considering sex, education level, histology, clinical staging and treatment in the three age groups. In univariate analysis, the prognostic factors for young individuals were education level, CS and treatment. In fact, complete elementary education and above (compared with incomplete elementary and illiteracy) were good prognostic factors, while advanced or metastatic disease (compared with early-stage disease – CSs I/II) was a poor prognostic factor in all three age groups. In addition, treatment with surgery (alone or in combinations) was a good prognostic factor for all age groups. Although sex and histological type were not prognostic factors for YA, male sex (compared with female) was a poor prognostic factor for MA and elderly patients, while adenocarcinoma histology (compared with squamous cell carcinoma) was a good prognostic factor in MA and elderly group (Table 3).

In multivariable analysis adjusted for age group, education, CS and type of treatment persisted as prognostic factors for all three age groups. Advanced stage and metastatic disease were poor prognostic factors compared with early-stage disease while surgery (alone or in combinations) was a good prognostic factor compared with other types of treatment for all the three age groups (Table 3). In addition, complete elementary education and above persisted as good prognostic factors for young and elderly and university education for MA patients. For MA and elderly individuals, male sex (compared with female) also persisted as a poor prognostic factor while adenocarcinoma histology (compared with squamous cell carcinoma) persisted as a good prognostic factor only for MA (Table 3).

We further evaluated the HR of death for young individuals within each variable category (Table 4) compared with other age groups. Hazards of death was lower for young males, as well as for young individuals diagnosed within all CS groups (early stage, advanced or metastatic disease), as compared with the older age groups. In addition, YA treated with surgery or CT had a lower HR of death than elderly patients submitted to the same type of treatment, but it was not different from MA people. On the other side, there was no difference in hazards of death within the main histological types (squamous cell, adenocarcinoma and large cells) for young patients compared with MA and elderly patients. For patients treated with surgery and CT, the HR of death was higher for elderly patients (Table 4).

## Discussion

The present analysis focused on a cohort of 17,422 patients registered with NSCLC in the period of 2000–2009 at the hospital-based cancer registry from Sao Paulo State, Brazil. According to the present results, the group of YA, aged 18–39 years, corresponded to 2.1% of the whole group of patients, half were men, 61.4% were diagnosed with adenocarcinoma and 63.2% presented with metastatic disease. Compared with older age groups (MA: 40–64 years and elderly (E): > 64 years), less YA were illiterate; more YA were women and more YA were diagnosed with adenocarcinoma. Surgery, RT and CT were offered to 23.2%, 38.4% and 71.6% of the young patients. Although more YA had metastatic disease at diagnosis, they presented a longer overall survival than patients in the other age groups.

The present results are in accordance with other authors, who have shown that lung cancer in YA is more commonly detected in women and that adenocarcinoma is more prevalent than in older patients [5, 11, 14]. In addition, more advanced CS at diagnosis seems to be a trend in young patients [19]. Accordingly, among patients aged 40 years or less, a greater proportion (63.2%, compared with 51.9% of MA and 43.2% of elderly patients, after excluding around 5% of the patients with undetermined CS) in the present series and 57.4% in a surveillance, epidemiology, and end results (SEER) study (compared with 43% of elderly individuals) were diagnosed with disease CS IV [5]. In a similar way, data from the Metropolitan Detroit Cancer Surveillance System, USA, showed that younger patients, less than 50 years, were less frequently diagnosed with local disease, i.e., 18.6%, compared with elderly individuals (25.2%) [4]. It seems likely young Brazilian patients are prone to present with metastatic disease at diagnosis. There is no clear explanation for this observation; however, lung cancer might have not been at first suspected, due to its rarity in young people, leading to a delay in diagnosis.

The median survival was quite poor in the three age groups, varying from 7.49 to 8.41 months. YA, in spite of being more frequently diagnosed with metastatic disease, lived longer than patients in older age groups. This effect was more pronounced in young patients diagnosed with disease CSs I/II and III, where a small proportion of individuals might reach durable remissions or even cure. This favourable outcome might be in part related with tumour genetics, as tumours from young patients more frequently harbour driver mutations that may be related with a better prognosis [20, 21]. In Brazil, data from a retrospective cohort study from the year 2014 indicate that around 50% of patients with metastatic adenocarcinoma were referred for molecular testing, among whom, 24% had a tumour with positive EGFR activating mutation and 87% received an EGFR-tyrosine kinase inhibitor. Patients harbouring EGFR mutation positive tumours had a longer median overall survival; however, it was not possible to individualise outcomes, according to patient age [22]. In this study, even though most of the patients were treated in the public health system, availability of target therapy is rather variable along the country to the present date.

Other factors that may suggest a differential carcinogenesis pathway in YA are a lower smoking burden in pack-years and a higher frequency of non-smoking females, when compared to older patients [9, 11]. In agreement, a recent study including 790 Brazilian lung cancer patients confirmed a less intense tobacco exposure in young patients (<55 years), detected by lower pack-years, compared with older age groups. However, the percentage of smoking patients was high, around 80% in young, as well as in the other two older age groups [23]. Despite that, the percentage of adult smokers in Brazil has dropped from 34.8% in 1989 to 12.6% in 2019, due to reinforcement of restriction laws [24]. Another factor that may have contributed to poor overall survival in older patients diagnosed with CS I or CS II is the presence of comorbidities [5]. In the present series, curative intent treatment, such as surgery, was more frequently offered to young and MA individuals diagnosed with early-stage disease. In addition, CT was more frequently offered to younger age groups diagnosed with metastatic disease. Unfortunately, elderlies tend to be less frequently exposed to more aggressive treatments due to related poor treatment outcomes, such as postoperative complications, CT intolerance and mortality, which may be related with frailty and may be observed in more than half of older cancer patients [25].

In accordance with the present results, improved outcome has been shown for young patients in studies analysing data from hospital-based cancer registries [14], as well as SEER studies [4, 5] and others [6]. Some studies have already described a more pronounced 5-year overall survival advantage at earlier CSs of the disease that decreased in more advanced stages, with absolute differences between the younger and older groups reaching 25% for stages I and II, but only 9% and 2% for stages III and IV, respectively [4, 5]. In contrast, there are other studies that do not corroborate the difference in survival between young and elderly patients [11, 26] (Supplementary Table 1).

In the present study, the level of education was inversely associated with mortality. In accordance, a study evaluating cancer mortality in the United States according to educational level and race [27] showed a 2.84 to 3.36 higher death rate among men with lung cancer with ≤12 years versus >12 years of education (for black and white ethnicities, respectively) and the same trend for women. In fact, the largest variability in cancer mortality associated with years of education was observed among lung cancer patients, when also considering cancer death rates for colorectal, breast or prostate cancer patients. This study, however, was restricted to individuals who were 25–64 years old at death. This is one of the main differences with the present study, as 48% of our population consisted of elderly people aged 65 years or more at cancer presentation. In addition, in our study, only a small part of the cohort, i.e. 7.2%, had 12 or more years of education.

The current results indicate that even though a higher proportion of young patients (37.1%) had education level above complete elementary grade than older age groups (MA: 23.4%; elderly: 14.8%), they (young) were more frequently diagnosed with metastatic disease. This data is in contrast with a previous study of our group, that showed an association between low education level and advanced stage of cancers [28]. A factor that may have contributed to this discrepancy is that, in this study, no consideration was given for the different age groups. In favour of our results, among young patients, other authors have already reported a greater proportion of metastatic disease at diagnosis [5]. In addition, education level has been increasing in Brazil along the years, indicating that higher education level is a trend in younger people [29], as we have observed. Thus, missing data for education level does not seem to have impacted the results.

The present data, however, may not reflect other regions of the country. São Paulo state, located in the southeastern region of Brazil, is the most populous state of the nation, with 43.93 million inhabitants (2018 estimate). The state presents an Human Development Index (HDI) of 0.833 and represents the third largest Gross Domestic Product (GDP) in Latin America in absolute figures, accounting for 32% of the national GDP [30–32].

In Brazil, every individual has the right to public health care and more than three-fourths of the Brazilian population benefit from the public healthcare system, Sistema Único de Saúde [33]. Cancer treatment in the public system is offered in comprehensive cancer care centres, called CACON, that offer treatment for all types of cancers, or in cancer care units, called UNACON, that offer treatment for the most prevalent cancers in Brazil, as well as associated General Hospitals, that perform oncological surgeries in the areas of Gynaecology, Mastology, Urology and Surgery of the Digestive System. São Paulo state cancer care network comprises 15 CACON, 50 UNACON, 6 general hospitals with oncological surgery and 3 RT specific units, distributed all over its territory [34]. All these public cancer units, as well some cancer care units of the private health care, contribute data to the hospital-based cancer registry run by FOSP, hence we may assume that the average population is well represented in the present series.

The strength of this study is the evaluation of a large database, summarising information from a large proportion of patients in a Brazilian state, with an organised health system. Limitations of the study include the absence of cancer specific survival data and information about smoking, family history, as well as comorbidities and molecular testing. Unfortunately, this data is neither available in other large databases in Brazil.

## Conclusion

In conclusion, in this Brazilian cohort of NSCLC patients, YA presented a longer survival than older individuals. Besides representing only 2.1% of the whole group, a great concern is that a large proportion, i.e. more than one half of the young patients presented with metastatic disease. As a large fraction of YA might present tumours harbouring driver mutations, usually associated with better outcomes, biological differences among NSCLC patients in different age groups may in part contribute to the abovementioned findings.

## Funding

This research did not receive any specific grant from funding agencies in the public, commercial or not-for-profit sectors.

## Conflicts of interest

The authors declare no conflicts of interest.

## Figures and Tables

**Table 1.** Characteristics of the study population.

Age group		Young 18–39 years	MA 40–64 years	Elderly ≥65 years	Total	*p*-value
		*n* = 370 (%)	*n* = 8,697 (%)	*n* = 8,355 (%)	*n* = 17,422 (%)	
Age at diagnosis	Mean (SD)	34.6 (4.5)	55.3 (6.3)	72.6 (5.6)	63.1 (11.2)	nd
	Median (min–max)	36 (30–39)	56 (40–64)	72 (65–99)	64 (20–99)	
Sex	Men	192 (51.9)	5,560 (63.9)	5,970 (71.5)	11,722 (67.3)	<0.001
	Women	178 (48.1)	3,137 (36.1)	2,385 (28.5)	5,700 (32.7)	
Education	Illiterate	5 (1.8)	408 (6.3)	935 (15.0)	1,348 (10.4)	<0.001
	Incomplete elementary	93 (33.8)	2,678 (41.0)	2,773 (44.6)	5,544 (42.6)	
	Complete elementary	75 (27.3)	1,913 (29.3)	1,584 (25.5)	3,572 (27.4)	
	High school	81 (29.5)	971 (14.9)	566 (9.1)	1,618 (12.4)	
	University education	21 (7.6)	558 (8.5)	357 (5.7)	936 (7.2)	
	Missing	95	2,169	2,140	4,404	
Histological type	Adenocarcinoma	227 (61.4)	3,926 (45.1)	3,388 (40.6)	7,541 (43.3)	<0.001
	Squamous cells	59 (15.9)	2,492 (28.7)	2,842 (34.0)	5,393 (31.0)	
	Large cells	8 (2.2)	209 (2.4)	189 (2.3)	406 (2.3)	
	Other unspecified	76 (20.5)	2,070 (23.8)	1,936 (23.2)	4,082 (23.4)	
CS	I–II	34 (9.2)	1,156 (13.3)	1,360 (16.3)	2,550 (14.6)	<0.001
	III	84 (22.7)	2,670 (30.7)	2,891 (34.6)	5,645 (32.4)	
	IV	234 (63.2)	4,516 (51.9)	3,611 (43.2)	8,361 (48.0)	
	Unknown	18 (4.9)	355 (4.1)	493 (5.9)	866 (5.0)	
Treatment	Surgery	86 (23.2)	1,856 (21.3)	1,460 (17.5)	3,402 (19.5)	<0.001
	RT	142 (38.4)	3,577 (41.1)	2,931 (35.1)	6,650 (38.2)	<0.001
	CT	265 (71.6)	6,000 (69.0)	4,863 (58.2)	11,128 (63,9)	<0.001

Note: Education: *p*-value considering the five categories of education in the three age groups (excluding missing cases); Treatment: *p*-value considering patients treated with surgery (yes versus no), RT (yes versus no) and CT (yes versus no); min: Minimum; max: Maximum; nd: Not done

**Table 2.** Median survival and probability of survival in 1, 2, 5 and 10-years.

		Median	Probability of cumulative survival (%)			
	Variable	Months	1-year	2-years	5-years	10-years
Age group	Young	8.41	42.5	22.8	11.2	9.0
	MA	8.51	39.0	20.1	8.5	5.0
	Elderly	7.49	35.7	17.5	6.2	2.4
	Total	7.98	37.5	18.9	7.4	3.8

**Table 3.** HR of death: univariate and multivariable analysis.

Univariate analysis for probability of death non-adjusted for age group							
		Young (*n* = 263)		MA (*n* = 6,325)		Elderly (*n* = 5,896)	
	Variable	HR (CI 95%)	*p*-value	HR (95% CI)	*p*-value	HR (95% CI)	*p*-value
Sex	Women	1		1		1	
	Men	1.11 (0.90–1.38)	0.327	1.24 (1.19–1.30)	<0.001	1.19 (1.13–1.25)	<0.001
Education	Illiterate	1		1		1	
	Incomplete elementary	0.57 (0.23–1.42)	0.230	0.95 (0.86–1.06)	0.372	0.93 (0.87–1.01)	0.076
	Complete elementary	0.34 (0.13–0.85)	0.021	0.84 (0.75–0.94)	0.002	0.83 (0.77–0.90)	<0.001
	High school	0.38 (0.15–0.96)	0.040	0.75 (0.66–0.84)	<0.001	0.82 (0.73–0.91)	<0.001
	University education	0.25 (0.09–0.68)	0.007	0.65 (0.57–0.74)	<0.001	0.68 (0.60–0.77)	<0.001
Histological type	Squamous cell	1		1		1	
	Adenocarcinoma	0.92 (0.68–1.24)	0.571	0.91 (0.86–0.95)	<0.001	0.90 (0.85–0.94)	<0.001
	Large cells	1.02 (0.46–2.24)	0.968	1.10 (0.96–1.28)	0.179	1.26 (1.08–1.46)	0.003
	Other unspecified	0.78 (0.54–1.12)	0.181	1.21 (1.14–1.28)	<0.001	1.11 (1.04–1.17)	0.001
CS	I/II	1		1		1	
	III	2.84 (1.65–4.90)	<0.001	2.27 (2.10–2.45)	<0.001	1.94 (1.81–2.07)	<0.001
	IV	5.63 (3.35–9.47)	<0.001	3.67 (3.40–3.96)	<0.001	2.80 (2.62–3.00)	<0.001
Treatment	Surgery (± CT; ± RT; ± RT and CT; ± Other combinations)	1		1		1	
	RT (± CT; ± Other combinations)	2.28 (1.66–3.13)	<0.001	2.23 (2.09–2.37)	<0.001	1.74 (1.63–1.86)	<0.001
	CT (± Other combinations)	2.35 (1.72–3.22)	<0.001	2.39 (2.23–2.55)	<0.001	2.04 (1.90–2.18)	<0.001
	Other combinations	1.25 (0.45–3.43)	0.671	4.05 (3.42–4.81)	<0.001	3.62 (3.11–4.21)	<0.001
	No treatment	7.78 (5.12–11.84)	<0.001	7.52 (6.94–8.15)	<0.001	5.30 (4.91–5.71)	<0.001

Multivariable analysis for probability of death adjusted for age group							
		Young (*n* = 263)		MA (*n* = 6,325)		Elderly (*n* = 5,896)	
	Variable	HR (CI 95%)	*p*-value	HR (95% CI)	*p*-value	HR (95% CI)	*p*-value
Sex	Women	1		1		1	
	Men	1.08 (0.84–1.40)	0.542	1.26 (1.20–1.33)	<0.001	1.22 (1.15–1.29)	<0.001
Education	Illiterate	1		1		1	
	Incomplete elementary	0.42 (0.16–1.06)	0.067	1.04 (0.94–1.17)	0.429	0.99 (0.91–1.07)	0.716
	Complete elementary	0.29 (0.11–0.76)	0.011	0.94 (0.84–1.05)	0.266	0.91 (0.84–0.99)	0.031
	High school	0.31 (0.12–0.81)	0.017	0.91 (0.80–1.03)	0.130	0.89 (0.80–0.99)	0.036
	University education	0.17 (0.06–0.49)	0.001	0.81 (0.71–0.93)	0.002	0.83 (0.73–0.94)	0.004
Histological type	Squamous cell	1		1		1	
	Adenocarcinoma	0.70 (0.48–1.02)	0.062	0.91 (0.85–0.97)	0.002	0.95 (0.89–1.01)	0.095
	Large cells	0.47 (0.11–2.05)	0.318	1.16 (0.97–1.39)	0.094	1.37 (1.15–1.63)	<0.001
	Other unspecified	0.89 (0.56–1.40)	0.610	1.11 (1.04–1.20)	0.003	1.04 (0.97–1.12)	0.257
CS	I/II	1		1		1	
	III	3.24 (1.65–6.37)	0.001	1.80 (1.63–1.99)	<0.001	1.70 (1.55–1.85)	<0.001
	IV	4.99 (2.54–9.81)	<0.001	2.86 (2.60–3.16)	<0.001	2.50 (2.29–2.73)	<0.001
Treatment	Surgery (± CT; ± RT; ± RT and CT; ± Other combinations)	1		1		1	
	RT (± CT; ± Other combinations)	1.58 (1.05–2.39)	0.027	1.66 (1.53–1.80)	<0.001	1.30 (1.19–1.42)	<0.001
	CT (± Other combinations)	1.56 (1.04–2.33)	0.030	1.71 (1.57–1.86)	<0.001	1.41 (1.29–1.55)	<0.001
	Other combinations	1.69 (0.48–5.94)	0.416	4.21 (3.32–5.34)	<0.001	3.94 (3.22–4.82)	<0.001
	No treatment	7.76 (4.26–14.16)	<0.001	5.43 (4.90–6.01)	<0.001	4.31 (3.91–4.76)	<0.001

Note: HR: Hazard ratio; 95% CI: 95% confidence interval

**Table 4.** Univariate analysis – HR of death for MA and elderly patients compared with young patients.

Variable		Age group	HR	95% CI	*p*-value
Sex	Male	Young			
		MA	1.168	1.005–1.357	0.043
		Elderly	1.269	1.092–1.475	0.002
	Female	Young			
		MA	1.053	0.898–1.236	0.522
		Elderly	1.199	1.021–1.406	0.027
Education	Illiterate	Young			
		MA	0.522	0.216–1.264	0.150
		Elderly	0.571	0.237–1.378	0.213
	Incomplete elementary	Young			
		MA	0.861	0.697–1.062	0.162
		Elderly	0.922	0.747–1.137	0.447
	Complete elementary	Young			
		MA	1.332	1.043–1.700	0.021
		Elderly	1.445	1.131–1.846	0.003
	High school	Young			
		MA	1.019	0.802–1.293	0.880
		Elderly	1.214	0.950–1.550	0.121
	University education	Young			
		MA	1.435	0.884–2.330	0.144
		Elderly	1.667	1.023–2.718	0.040
Histological type	Squamous cell	Young			
		MA	1.039	0.793–1.361	0.781
		Elderly	1.185	0.906–1.553	0.215
	Adenocarcinoma	Young			
		MA	1.005	0.874–1.155	0.944
		Elderly	1.135	1.135–0.987	0.076
	Large cells	Young			
		MA	1.116	0.524–2.378	0.775
		Elderly	1.388	0.650–2.963	0.397
	Other unspecified	Young			
		MA	1.588	1.240–2.034	<0.001
		Elderly	1.653	1.290–2.117	<0.001
CS	I–II	Young			
		MA	2.021	1.234–3.319	0.005
		Elderly	2.991	1.826–4.901	< 0.001
	III	Young			
		MA	1.511	1.191–1.916	0.001
		Elderly	1.832	1.444–2.323	< 0.001
	IV	Young			
		MA	1.147	1.005–1.310	0.042
		Elderly	1.238	1.084–1.415	0.002
	Surgery	Young			
		MA	1.208	0.937–1.557	0.145
		Elderly	1.512	1.171–1.951	0.001
Treatment	RT	Young			
		MA	0.977	0.823–1.160	0.793
		Elderly	0.984	0.828–1.168	0.851
	CT	Young			
		MA	1.108	0.975–1.258	0.117
		Elderly	1.176	1.034–1.336	0.013

Note: HR: Hazard ratio; 95% CI: 95% confidence interval

Treatment: Patients treated with surgery (yes versus no), RT (yes versus no) and CT (yes versus no)

**Supplementary Table 1.** Overall survival analysis in young patients.

Study	No. of patients	No. of young patients	Cut off age	Histology in young (%)	Histology in elderly (%)	Survival analysis	Survival in young	Survival in elderly	*p*-value
Arnold *et al* [14](USA)	173,856	5,657	46	A: 49.2 S: 15.7 L: 5.7 O: 29.3	A: 39.2 S: 29.9 L: 4.1 O: 26.8	5-years	CS I: 70% CS II: 49% CS III: 22% CS IV: 5%	CS I: 45% CS II: 26% CS III: 13% CS IV: 3%	<0.0001
Subramanian *et al* [5] (USA)	239,088	2,775	40	A: 57.5 S: 12.5 L: 10.3 O: 19.7	A: 45.2 S: 26.4 L: 7.6 O: 20.8	5-years	Higher survival in young patients in all CS comparing with elderly		<0.001
Mauri *et a*l [26] (Greece)	1,906	115	45	A: 48.7 S: 23.5 L: 10.4 O: 15.7	A: 42.7 S: 36.5 L: 54.9 O: 11.7	Median	12 months (10.5–13.1)	11.5 months (10.9–12.2)	0.277
Jiang *et al* [8] 2012 (China)	3,320	626	45	A: 37.7 S: 21.1 L: 0.3 SC: 36.6 O: 4.3	A: 35.8 S: 39.4 L: 0.1 SC: 21.1 O: 3.6	1-year 3-years 5-years	62.73% 23.97% 13.75%	66.29% 30.71% 17.53%	0.0232
Radzikowska *et al* [6] (Poland)	5,404	757	50	A: 12.6 S: 34.9 SC: 22.9 O: 29.6	A: 7.6 S: 42.0 SC: 14.8 O: 35.7	1-year	32.60%	28.90%	<0.049
Tominaga *et al* [11] (Japan)	790	77	50	A: 78.9 S: 10.5 L: 2.6 SC: 7.9 O: 1.3	A: 49.4 S: 35.1 L: 2.7 SC: 12.9 O: 2.0	2-years 5-years	41.3% 33.0%	41.8% 33.1%	0.936 0.769
Ramalingam *et al* [4] (USA)	31,266	2,813	50	A: 44.4 S: 26.7 L: 10.4 SC: 17.4	A: 33.7 S: 37.9 L: 7.4 SC: 21.0	5-years	16.10%	13.40%	<0.0001

Note: CS: Clinical stage; m: Months; No.: Number; Histology: A – Adenocarcinoma, S – Squamous cell carcinoma, L – Large cell carcinoma, SC – Small cell carcinoma, O – Other; Y: Years
